# Availability, Use, and Cultivation of Support Networks as Predictors of the Well-Being of Middle-Aged and Older Chinese: A Panel Study

**DOI:** 10.1100/2012/978036

**Published:** 2012-05-03

**Authors:** Alice Ming Lin Chong, Chau-kiu Cheung, Jean Woo, Alex Yui-Huen Kwan

**Affiliations:** ^1^Department of Applied Social Studies, City University of Hong Kong, Tatchee Avenue, Hong Kong; ^2^Department of Medicine and Therapeutics, The Chinese University of Hong Kong, Hong Kong

## Abstract

*Objectives*. To examine the impact of the availability, use, and cultivation of a support network on the well-being of community-dwelling, middle-aged, and older Chinese. *Methods*. A total of 2,970 Hong Kong Chinese aged 40–74 years were interviewed using a structured questionnaire in 2004. Out of the original group of interviewees, 2,120 (71.4%) were interviewed again in 2005. *Results*. Structural equation modeling revealed a good fit of the model employing Wave 1 support network data and demographic characteristics to predict Wave 2 well-being. As hypothesized, the availability of important social ties and the cultivation of one's support networks were found to predict well-being one year later, but not the use of support networks to meet emotional, financial, or companion needs after controlling for demographic variables and baseline well-being. *Discussion*. Cultivating support networks can be interpreted as positive and active coping. Such cultivation is in line with what socioemotional selectivity theory predicts; specifically, when people age, they become more selective and concentrate on strengthening their relationship with those they are emotionally close to. We argue that network cultivation deserves more attention in theory, practice, and research to strengthen the resilience and adaptability of individuals approaching and experiencing old age.

## 1. Introduction

Social support is associated with well-being in old age, including better mental and physical health [1–3] and satisfaction with life [4], as well as a lower likelihood of depression [5], anxiety [6], dementia [7], and mortality [8]. It also buffers the adverse effect of stress, and facilitates positive or successful aging [9, 10]. Lack of support during stressful times brings about adverse effects such as heart dysfunction [11]. On the other hand, other studies have found limited or even negative effects of social networks [12, 13].

Existing studies usually focus on the size of support networks [2, 4, 14, 15]. However, the existence of support networks, as indicated by their size, does not always translate to actual *use* when the need arises. Another important but overlooked issue is whether or not individuals exert effort to *cultivate* their significant others or their support networks, to maintain or replenish old networks, and to expand new networks that may strengthen their resilience and adaptability in old age.

To answer these questions, a panel study of 2,970 community-dwelling Hong Kong Chinese aged 40–74 years was carried out in 2004 and 2005. The objective was to test the interplay between the availability, use, and cultivation of social support networks, and to discover the effects of network cultivation on well-being as measured by positive aging components. In addition, this study addresses the relative neglect of midlife in aging research. It examines aging during and from midlife to provide a wider life-span perspective in identifying age-related changes and social network strategies that strengthen an individual's resilience and adaptability to aging. Midlife is a time when there are opportunities for meaningful intervention and preparation for successful aging [16]. Moreover, this panel study presents two waves of data collected over one year and makes it possible to examine the extent to which baseline support network components can predict well-being one year later.

## 2. Literature Review and Hypothesis

The term “social support” and “support networks” are often used interchangeably and can be defined as “an interrelated group of people, resources, and organizations that provide individuals with emotional, informational, materials, and affectional sustenance” [17], or as information from others that one is loved and cared, esteemed and valued, and part of a network of communication and mutual obligations [18]. Social support has been established as one of the most effective resources to facilitate coping and adjusting to stressful events, thereby buffering individuals from the adverse mental and physical health effects of stress [18, 19] prominent in old age [2, 4].

Well-being, the second key aspect of the present paper, has been the subject of numerous studies. According to the literature on successful aging, well-being in late life has been thought of in terms of physical health, functional independence, and active social engagement [9]. However very few studies have been found on social engagement [20], in contrast to the abundant studies conducted on health and functioning abilities. One of the few studies on ways to age well has been carried out by the authors [21]. In the study, 15 groups of middle-aged and older Hong Kong Chinese identified the themes of good health, independent functioning, desire to continue to care for the well-being of younger generations, and to remain useful to the society. The participants' discourse on social engagement reflects the Chinese cultural meanings of social engagement in late life, which point to two equally important but distinct aspects that correspond roughly to social-emotional and instrumental-productive engagements. *Caring* engagement with others by showing care and love constitutes a major form of social engagement in Chinese culture [22], and is grounded on the Confucian ethic of caring for young ones and for elders in the family as well as in other families. The second aspect is *productive* engagement. This aspect is grounded on the Chinese folk belief that “elders have roles to play” which exhorts elders to continue contributing to society, and is firmly entrenched in the Confucian ethics of diligence and social contribution [23].

Taken together, aging well goes beyond the avoidance of illness and continuous proper functioning, but also to social well-being as a caring and productive member of one's family, community, and society. Ng and his colleagues [24] have recently differentiated Rowe and Kahn's [9] single social engagement component into a caring (CE) and a productive (PE) component, which were empirically confirmed by data collected from 233 middle-aged and older adults in Hong Kong through a validation study. The study demonstrated the abilities of CE and PE (as well as the two health components) to discriminate adults who aged well from those who aged less well.

Based on prior studies on support networks and well-being in late life, three hypotheses form the guidepost of the present study.

**Hypothesis 1**

Network availability in Wave 1 will positively predict well-being components in Wave 2.

Network availability refers to the size of active networks, namely, the number of people whom the respondents feel close to or are important to them. Network size has often been found to be associated with well-being in old age [25, 26]. However, recent evidence has found a negative effect of network size on well-being [2] and on emotional closeness [27], a distinct upper bound on total network size [27]. Moreover, the evidence points to the benefits of selective diminution of network ties late in life [28]. Therefore, it is important to identify the direction of network availability prediction, if any, on well-being starting from midlife.

**Hypothesis 2**

The use of personal support networks negatively predicts well-being components one year later.

While some studies have found that giving and receiving support from others have positive and negative consequences [29], other studies have shown that receiving help from network members correlates with lesser well-being [30]. Thus, it appears that the use of social support on well-being is not conclusive. The predicted negative association is also derived from recent studies on cultural influence and its effect on how individuals seek, obtain, and benefit from their close associates. For example, receipt of money was negatively associated with depressive symptoms among older people living in non-Mediterranean countries, but was positively related among their Mediterranean counterparts, possibly because receipt of money from children is accompanied by a sense of shame in the Mediterranean context [13].

Studies [18, 31] have found that Asians and Asian Americans are more reluctant to ask explicitly for help from close associates compared to Europeans and Americans. In collectivistic cultures, the self is viewed as interdependent, and individuals are more concerned about the potentially negative relational consequences of help seeking, such as burdening their close associates and losing face. In comparison, in individualistic cultures, relationships are viewed as freely chosen and entail relatively fewer obligations [18]. In addition, verbal sharing of thoughts and feelings are emphasized and frequently exercised. Explicit social support will therefore be more commonly used and seen as beneficial [31]. In this light, the Chinese and its collectivistic culture might not regard seeking of social support as beneficial.

**Hypothesis 3**

Network cultivation in Wave 1 positively predicts positive well-being components in Wave 2.

Very little studies have been conducted on network cultivation. Cultivating one's relationships with kin and nonkin implies taking the initiative and exerting effort to strengthen relationships with people they already know. All these call for positive motivation and an active approach in network behavior, and can count as a form of coping strategy to enable individuals to build their social resources in preparation for the different challenges and stresses in life [18]. It is therefore hypothesized that participants who exert effort to cultivate their close ties would have better well-being due to their positive and active coping orientation.

## 3. Methods

The current panel study was conducted in 2004 (Wave 1) and 2005 (Wave 2) through face-to-face interviews by trained interviewers who were undergraduates in the social sciences. The interviews were carried out at either the interviewees' home or in venues nominated by the interviewees themselves. A few interviews (.6%) also took place over the phone at the interviewees' request. The interval between each pair of interviews was 12 months. Random checking of 10% of each interviewer's interviews by the research team was undertaken for quality assurance. More details on the study method, which includes sampling, can be found in another article written by the authors [24].

### 3.1. Participants

The sampling criteria were Hong Kong Chinese 40–74 years of age, living in the community, and able to communicate verbally with the interviewers. A crucial sampling criterion was the participants' willingness to respond to the survey again one year later. Therefore, a special sampling method was designed to achieve this. In Wave 1, a total of 2,970 participants were recruited by 68 paid undergraduate interviewers who each nominated 40 adults who met the sampling criteria. The nominees' names were assigned to *another* undergraduate for interviewing. The goal of the process was to interview 800 adults each for the three younger age groups: 40s, 50s, and 60s. For participants aged 70–74 years, the target number was increased from 400 (half of 800) to 500. The increase would serve as a buffer in case there was difficulty contacting the original group of participants for the follow-up survey. The overall nonresponse rate was less than 8%. Twelve months later, the follow-up survey was conducted with 2,120 of the previous year's participants, a successful retention rate of 71.4% [24]. After each interview, the participant received a supermarket coupon of HK$100 (or US$12.8) in acknowledgment of his or her contribution to the study.

### 3.2. Measures

The personal support network was measured by a specially constructed 23-item Support Network Scale (SN Scale). The SN Scale is composed of three theoretical social network components, namely, network availability, use, and cultivation, which were developed along the findings of a qualitative study on 15 focus groups of middle-aged and older Hong Kong Chinese [21]. The SN-Availability Subscale (5 items) explored the size of active social networks among family, relatives, friends, and neighbors, and provided an overall assessment. For example, respondents were asked, “How many of your family members do you feel close to?” and had to choose from among five options, ranging from 1 = none, 2 = 1-2 people, 3 = 3-4 people, 4 = 5-6 people, and 5 = more than 6 people.

The SN-Use Subscale (14 items) measured the extent to which the respondents utilized their various support networks of family, relatives, friends, neighbors, and others (such as social workers, social institutions, etc.) when experiencing different emotional, financial, and relational needs. Five items were concerned with seeking emotional support, such as the participants being asked if they would seek help from their family during emotional problems. Another five items were about seeking financial support during economic hardship while four items explored the extent to which participants sought company for social leisure activities. The SN-Cultivation Subscale (4 items) examined the amount of time and effort the respondents devoted to cultivate their relationship with family, relatives, friends, and neighbors. All items on the SN Scale adopted a five-point rating scale, with 1 being the lowest value and 5 as the highest value. The overall score of each subscale was the mean of all items in the relevant subscale.

The Cronbach's alpha coefficients were  .82, .63, .75, and  .60 for the overall SN Scale, SN-Availability Subscale, SN-Use Subscale, and SN-Cultivation Subscale, respectively, in Wave 1. The coefficients slightly improved in Wave 2 at  .83,  .65,  .78, and  .64, respectively, indicating good reliability in the overall scale and moderate reliability in the three subscales.

Well-being components were measured by the Positive Aging Index (PAI), which was created by the research team. The index has good psychometric properties based on a validation study of 233 Chinese adults (40–90 years) and a longitudinal study of 2,970 Hong Kong Chinese [24]. The PAI extended Rowe and Kahn's three-factor model of successful aging into four factors: health, functioning, caring engagement (CE), and productive engagement (PE). The longer (18 items) and shorter (12 items) versions were equally valid and reliable, and the shorter version was used in this study. Health (3 items) covered bodily pain, physical mobility, and an overall self-assessment of physical health. Functioning (3 items) comprised energy level, sleep, and ability for independent living. CE with life (4 items) was about care and support shown to family, relatives, and friends, followed by an overall self-assessment of how concerned and supportive the respondents were. PE (2 items) measured tangible or productive contributions made to the community and the society. A five-point rating scale was used, and the overall score of the scale was the average of all items. The Cronbach's alpha coefficients were  .75,  .62,  .61,  .76, and  .61 for overall PAI, health, functioning, CE, and PE, respectively, in Wave 1. These values slightly improved in Wave 2 at  .76,  .67,  .66,  .72, and  .64, respectively, suggesting satisfactory reliability of the overall scale and the subscales. The Cronbach's alpha coefficients of the subscales were not high possibly due to the small number of items [32]. The following demographic characteristics of the respondents were also asked in the questionnaire: gender, age, years of education, marital status, and household income. Dummy variables were created for gender (female = 1) and marital status (currently married = 1).

### 3.3. Data Analysis

Structural equation modeling via M*plus* [33] estimated the effects of Wave 1 predictors, including support network components and background characteristics, on the four Wave 2 well-being outcomes in a holistic way. The modeling allowed for an examination of the goodness of fit of the whole model. It also constrained the standardized effect of each component of the three support networks (availability, use, and cultivation) on the four well-being components (health, functioning, CE, and PE) to be equal. That is, the examination showed the goodness of the model with the constraints.

## 4. Results

### 4.1. Participant Characteristics

The 2,970 respondents interviewed in Wave 1 were stratified into the following four age groups: 40–49, 50–59, 60–69, and 70–74. Their percentages were 39% (873), 27% (811), 26% (771), and 17% (515), respectively. Majority of the participants were women (62.3%), married (78%), living with spouses only or living with spouse together with others (74.4%), with 1–3 children (67.8%), engaged in gainful employment (40.6%), and had received primary education or below (65%). In the Wave 2 survey, the respective numbers were 609 (40 s), 584 (50 s), 553 (60 s), and 374 (70–74 years) for a total of 2,120 respondents. The demographic profile of the participants in Wave 2 was very similar to that of Wave 1. When compared with the census data [34], the sample was comparable to the corresponding age groups in the population, except for the underrepresentation of men and those in higher income groups.

### 4.2. Prevalence of Availability, Use, and Cultivation of Support Networks

Table 1 shows the means and standard deviations of the support network components in Waves 1 and 2, and the results of the paired samples *t*-test. The participants' overall support network fell slightly below the scalar midpoint of 3 (Wave 1 *M* = 2.65, SD = .51; Wave 2 *M* = 2.67, SD = .51). From the three support network components, participants had the highest score in SN-Availability (*M* = 3.23, SD = .82), followed by SN-Cultivation (*M* = 2.93, SD = .69), and network use (*M* = 2.34, SD = .53) which had the lowest score. Paired samples *t-*tests found no significant difference in scores of the overall support network and the various network components between Waves 1 and 2. The only exception was the use of network, which was slightly higher in Wave 2 (*P* < .01). The general similarity in the scores of network components between Wave 1 and Wave 2 suggests that support networks are relatively stable among the participants. The frequency distributions of SN subscales at Wave 1 are reported in the following paragraphs.

### 4.3. Well-Being and Its Relationship with Support Network Components

Table 1 shows that the mean score of the PAI was 3.60 (SD = .51) in Wave 1 and 3.56 (SD = .49) in Wave 2, reflecting a good degree of overall well-being in the two periods. Among the four components of well-being, participants had the highest score in functioning (*M* = 3.96, SD = .72), followed closely by health (*M* = 3.95, SD = .76) and CE (*M* = 3.54, SD = .72). They had a relatively low score in PE (*M* = 2.63, SD = .89). Paired samples *t*-test found significant differences in both waves for overall well-being and its components of health and functioning.

The model using Wave 1 variables to predict Wave 2 well-being components attained a very good fit (*L* ^2^(9) = 44.1, SRMR = .010, RMSEA = .036, CFI = .996). Among the Wave 1 variables, support network availability (*β* = .031) and support network cultivation (*β* = .055) had significant positive effects on the four well-being components (Table 2). Note that well-being and sociodemographic characteristics had been controlled under Wave 1 and that the effects were equal on the four well-being components. The significant effects, although very weak, supported Hypotheses 1 and 3. On the other hand, the effect of the use of support networks on well-being components was not significant, and Hypothesis 2 was not supported. 

The other significant predictors of Wave 2 well-being were Wave 1 well-being, gender, age, education, marital status, and family income (Table 2). The findings indicated some continuation of well-being. Moreover, one component of well-being could be predictive of another component of well-being one year later. Women scored lower in health and functioning but scored higher in CE compared to men. Health, functioning, and CE declined with age. Higher CE was associated with education, marriage, and family income. Education also contributed to PE. The currently married person was lower in functioning compared to another person who was divorced, separated, or not married. Altogether, the Wave 1 predictors explained the 22–27% variation of the four components of well-being (*R* ^2^ = .221–.272).

## 5. Discussion and Recommendations

The objective of this study was to examine the predicting effect of support network components (availability, use, and cultivation) on well-being among community-dwelling middle-aged and older people one year later. A panel study was conducted through face-to-face interviews with 2,920 Hong Kong Chinese aged 40–70 in 2004 (Wave 1); 71% of whom were interviewed again one year later (Wave 2). 

Results show that, in general, the participants had a high level of well-being in terms of health (low level of pain, able to move around freely, and enjoying good overall health) and functioning (having enough energy, able to sleep well, and living independently). They were found to be below scalar midpoint in terms of PE in the community and the society as a whole. Therefore, greater effort is required to enhance the PE of older people (such as volunteering) and to promote proper recognition of their contributions (such as household chores and informal care giving) which are commonly delegated to older people. 

### 5.1. Network Availability Predicted Well-Being One Year Later

The availability of active networks was found to predict overall well-being one year later, even after controlling for personal variables and Wave 1 well-being. Thus, the first hypothesis that network availability in Wave 1 will positively predict well-being and its components in Wave 2 was supported. 

Network availability carried a positive prediction on well-being. This finding implies that the larger the network size, the higher the well-being. Nevertheless, the mean score of SN-Availability (*M* = 3.23, SD = .82) implies that, on average, the participants felt close to 3 to 4 persons among family members, friends, relatives, or neighbors. The finding supports the Roberts and associates' [27] argument that the size of active networks would be modest due to cognitive issues (e.g., keeping track of a large number of relationships simultaneously) and/or time budget issues (e.g., the time to build the relationship). 

### 5.2. Why Was Well-Being Not Predicted by Network Use?

The second hypothesis was that the use of personal support networks would negatively predict well-being one year later. The present study found that the use of personal networks for emotional, financial, and social support did not predict the participants' well-being one year later. 

Support networks serve different functions; some are associated with pleasant experiences such as seeking companionship in social-recreational activities. In contrast, support networks within the context of seeking help for financial and emotional problems are negative by nature. Even if some significant others help, there is concern over reciprocity and incurring a sense of indebtedness which do not contribute to a sense of well-being. This lack of association between seeking support and well-being is partly explained by cultural concern on the negative relational consequences of help seeking in a collectivistic culture [18]. Social support is contextual and culture bound; it is viewed in relation to the social values and expectations of the older people [13]. In Chinese culture, it is stipulated that a person should be self-sufficient; thus, seeking help may be associated with failure. 

### 5.3. Network Cultivation Should Be Encouraged

Cultivation of support networks predicted well-being one year later, supporting the third hypothesis. The greater the efforts exerted by middle-aged and older participants in cultivating their social relationships, the greater their possibility of achieving better well-being. Most likely this is due to a higher likelihood of establishing a social network that is enduring and ready to provide various kinds of support when needed. Nevertheless, it was found that network use cannot predict well-being one year later. Taken together, these results suggest that the positive effect of network cultivation might come more from positive coping rather than the reassurance of supportive networks or the actual use of the networks. 

For older people, coping strategies are frequently applied when they are confronted with aging-related problems and obstacles [35]. Cultivating support networks can reflect a positive, active, and approach-oriented coping strategy. These tend to be more effective than avoidance or passive coping methods under normal situations. Further studies are recommended to look into the contribution of an individual's effort to cultivate their social ties as a coping strategy. 

The finding provides some support to the socioemotional selectivity theory [36, 37], which suggests that as people advance in age, they become more conscious of future time and become more selective. They concentrate on strengthening and maintaining their relationships with a few who are important to them or who possess higher socioemotional importance. 

### 5.4. Support Networks Are Associated with Well-Being

Similar to other studies that established social relations as an important determinant of well-being [38, 39], the present study found that the size of close ties with kin and nonkin predicted positively the respondents' different aspects of well-being (including health, functioning, CE, and PE) one year later. The two important contributions of the present study are the cultivation of social ties as a predictor of well-being and the positive effects of support networks carried forward one year later. However, the variance explained by these variables on the relevant well-being outcomes was very low, which implies that other variables can explain the variance. In Table 2, well-being at the baseline was a stronger and more statistically significant predictor of well-being than support networks one year later. These temporal associations suggest that good health and functioning, active engagement with significant others, and contribution to others and society bring about a sense of well-being here and now. They also provide a solid foundation for well-being in the future. 

### 5.5. Limitations of the Study

This study has several limitations. First, the sampling approach was not random, thus limiting the generalizability of the findings. Nevertheless, the sample was large enough to reflect the major views of middle-aged and older Chinese people in Hong Kong. Moreover, a year's worth of panel data was too short to identify significant changes in social networks and well-being over time. The measure of social support relied on self-reports that reflect intention or attitudes rather than actual behavior. Participants may behave differently in real-life situations. 

## 6. Conclusion

This panel study, conducted over a one-year period, found that among the three social network components, cultivating relationship with significant others had the strongest predicting effect on well-being. This was followed by the size of available support networks. The use of support networks to meet various financial, social, and emotional needs did not predict well-being. This illustrates that middle-aged and older people age well if they had a relatively large number of kin and nonkin members with whom they felt close, and if they had taken efforts to cultivate their relationships with their significant others. Whether they used their support networks for help or support was not related to their sense of well-being. This is possibly due to the cultural reservations associated with help seeking such as losing face, unduly being a burden on others, and the cultural emphasis on self-sufficiency. Cultivating support networks also reflected a positive and approach-oriented coping strategy. 

These findings carry important theoretical and practice implications. The findings lend support to the socioemotional selectivity theory, which argues that when people age, they become more selective and concentrate on strengthening their relationship with those they feel close to. The findings also support the argument to include caring and productive engagement in addition to health and functional abilities in the successful aging framework. In practice, human service professionals like social workers should enhance older people's people skills, such as skills to communicate with and engage their significant others, as well as skills in the use of modern communication technologies such as email and Facebook to communicate with the younger members of the family. In traditional Chinese society, older people enjoy high status and seldom take the initiative to build relationships with their family members. It is the younger generation who actively pays respect to the senior members of the family. Therefore, human service professionals should encourage senior citizens to take active roles in building and sustaining their relationships with their significant others. 

As far as we know, the present study is one of the very few attempts to examine an individual's active efforts in network cultivation. We argue that network cultivation deserves more attention in theory building, further studies, and human service practice to strengthen the resilience and adaptability of individuals approaching and experiencing old age. While this study was conducted in the Chinese community of Hong Kong, the findings may provide some useful reference to other Chinese societies in Shanghai, Singapore, Taiwan, and Chinese immigrant communities in the West. Many of the older people in these societies were born in their homelands and migrated to different societies in their youth due to wars, famine, and economic hardship. They may share similar childhood experiences, developmental experiences, and value systems with those of Hong Kong Chinese. 

## Figures and Tables

**Table 1:** Paired samples *t*-test of social network components and well-being components of Wave 1 and Wave 2 (*N* = 2, 120).

	Wave 1 (*N* = 2, 970)		Wave 2 (*N* = 2, 120)		*t-*test
	*M*	SD.	*M*	SD.	t
Support Network Scale					
Support network availability	3.23	.82	3.20	.80	1.81
Support network use	2.34	.53	2.37	.53	−2.98**
Support network cultivation	2.93	.69	2.93	.71	−.05
Overall Support Network Scale	2.64	.51	2.65	.50	−1.38
Positive Aging Index^∧^					
Health	3.95	.76	3.90	.76	2.78**
Functioning	3.96	.72	3.88	.70	5.25***
Caring engagement	3.54	.72	3.53	.64	.82
Productive engagement	2.63	.89	2.61	.87	.93
Overall positive aging	3.60	.51	3.56	.49	4.09***

*P < 0.05 (2-tailed), **P < 0.01 (2-tailed), ***P < 0.001 (2-tailed).

Scaling format of all scales: 5-point Likert scale, ranging from 1 (lowest value) to 5 (highest value).

^∧^Positive Aging Index: 12-item short version.

**Table 2:** Standardized effects on well-being at Wave 2 (*N* = 2, 120).

Predictor	Health	Functioning	Caring engagement	Productive engagement
Health, Wave 1	.324***	.163***	.047*	.035
Functioning, Wave 1	.166***	.320***	.036	.003
Caring engagement, Wave 1	−.009	−.011	.296***	.111***
Productive engagement, Wave 1	.045*	.026	.066*	.378***
Support network availability, Wave 1	.030*	.030*	.031*	.031*
Support network use, Wave 1	−.014	−.014	−.014	−.014
Support network cultivation, Wave 1	.055***	.055***	.055***	.055***
Female	−.085***	−.077***	.096***	.028
Age	−.119***	−.089***	−.074***	−.006
Education	.019	.011	.044*	.058**
Currently married	−.033	−.061**	.056**	−.009
Family income (logged)	.016	.021	.071**	.039
*R* ^2^	.272	.240	.221	.237

*P < .05. **P < .01. ***P < .001.
